# *Sphagnum* moss and peat comparative study: Metal release, binding properties and antioxidant activity

**DOI:** 10.1371/journal.pone.0307210

**Published:** 2024-08-19

**Authors:** Maria Luisa Astolfi, Maria Agostina Frezzini, Lorenzo Massimi, Mattia Rapa, Silvia Canepari, Marcelo Enrique Conti

**Affiliations:** 1 Department of Chemistry, Sapienza University of Rome, Rome, Italy; 2 Research Center for Applied Sciences to the Safeguard of Environment and Cultural Heritage (CIABC), Sapienza University of Rome, Rome, Italy; 3 ARPA Lazio, Regional Environmental Protection Agency, Rome, Italy; 4 Department of Environmental Biology, Sapienza University of Rome, Rome, Italy; 5 C.N.R. Institute of Atmospheric Pollution Research, Monterotondo St., Rome, Italy; 6 Department of Management, Sapienza University of Rome, Rome, Italy; Universitat Jaume 1, SPAIN

## Abstract

Peat is the main constituent of cultivation substrates and a precious non-renewable fossil material. Peatlands provide important ecosystem services and allow the absorption and storage of carbon. Protecting peatlands helps tackle climate change and contributes to biodiversity conservation. Due to its importance, it is necessary to implement strategies to reduce the use of peat, such as replacing it with biomass-based alternative growing media constituents, such as *Sphagnum* moss. In this study, we compared the metal release and binding properties at two different pH, antioxidant activity, and total phenolic content of peat and *Sphagnum* moss from the Tierra del Fuego (TdF) region of southern Patagonia. Levels of the elements were determined by inductively coupled plasma mass spectrometry (ICP-MS), while the types and amounts of functional groups were characterized and compared using Fourier transform infrared (FTIR) spectroscopy. The total phenol level and antioxidant capacity were assessed using the Folin-Ciocalteu method and 2,2-diphenyl-1-picrylhydrazyl test. There are generally higher concentrations of leachable elements in peat than in *Sphagnum* moss at pH = 2, except Cs, Rb, Ti, and Zr. In contrast, at pH = 5, levels of all leached elements are highest in *Sphagnum* moss. *Sphagnum* moss shows a higher metal adsorption capacity than peat, except for Be, Mn, Tl, and Zn. Finally, the results showed that both matrices contained similar total phenolic contents: 0.018 ± 0.011 mg gallic acid equivalent (GAE) per gram dry sample for peat and 0.020 ± 0.007 mg GAE g^-1^ for *Sphagnum* moss. Instead, *Sphagnum* moss extracts showed a significantly higher antioxidant activity [0.026 ± 0.028 mmol Trolox equivalents (TE) g^-1^] than that estimated in peat (0.009 ± 0.005 mmol TE g^-1^). Humic acids, carboxylic acids, and phenolic and lignin groups were identified as the functional groups that mainly determined the antioxidant activity of the *Sphagnum* moss compared to peat. The present study resulted in an advancement of knowledge of these materials for more thoughtful future use and possible replacements.

## Introduction

Peat is a complex organic matrix formed due to the slow and continuous accumulation of plant residues in depressions in the ground where water and humidity collect and certain climatic and environmental conditions occur [1, 2]. Peat is considered a natural heritage whose formation process occurred over millions of years. For this reason, it can only be extracted from natural deposits and not produced artificially. Peatlands exert a dual influence on climate change, exhibiting both positive and negative effects. Peatlands help regulate the climate by storing carbon in the peat and thereby reducing carbon dioxide from the atmosphere [3, 4]. Peat is also the main constituent of horticultural growing media [3, 5] but its extraction process generates greenhouse gas emissions [6]. Furthermore, peat extraction potentially threatens ecosystems and biodiversity [7]. In a context of growing awareness regarding the importance of protecting peat bogs and the need to take actions to combat climate change [8–11], in recent years attempts have been made to reduce the use of peat in growing substrates and to replace it with alternative products, such as green compost, wood fibers, and composted bark and coconut [6, 12–14]. In particular, the cultivation of *Sphagnum* sp. with paludiculture could represent further potential for replacing peat [13].

There are 16 elements (B, C, Ca, Cl, Cu, Fe, H, Mg, Mn, Mo, N, O, P, K, S, and Zn) without which plants could not grow and reproduce normally [15, 16]. However, plants can absorb other environmental elements, which can be toxic or potentially toxic to humans [17]. The absorption of nutritional elements depends on various factors, such as the effectiveness of absorption of individual nutrients and specific needs of the plant species, properties of the soil, such as pH, amount of organic matter, P content in the soil, and climatic conditions. In particular, peat and *Sphagnum* moss are natural bioabsorbents capable of binding some elements and gradually releasing them to the plants according to the chemical balances that are established. The metal binding properties are due to the presence of cellulose, lignin, and organic acids (such as humic and fulvic acids), and, therefore, through the presence of numerous active functional groups (such as phenolic, sulphonic and carboxyl) capable of absorbing metal ions through different types of chemical interactions, such as complexation, adsorption, and ion exchange [18, 19].

In addition, the preservative properties of *Sphagnum* moss and peat have been known since ancient times [20]. The products of the phenolic decomposition of mosses and other compounds contained in soils and peat have exhibited antioxidant activity and the ability to protect the biodegradation of soil organic matter through oxidation [21–24]. For example, compounds in soil, peat and plants that have shown antioxidant activity are humic acids [25–27], amino acids [28], lipids [29], peptides [30–32], and lignin [33]. The mechanisms of action of antioxidants depend on their chemical structure [34–37]. They are related to their ability to neutralize free radicals directly [38] and chelate transition metals [39, 40]. It is also necessary to consider possible synergies in interactions between different antioxidants [41, 42]. The antioxidant capacity of soils can also contribute to the conservation of microbial biodiversity and can be used as an indicator of soil health and quality [30]. The antioxidant capacity also affects the mineralization process of peat [21].

Given the importance of reducing the use of peat to limit greenhouse gas emissions and maintain biodiversity, this study aims to compare peat and *Sphagnum* moss in terms of leachable element content, metal binding properties, antioxidant capacity and total phenolic content to increase knowledge on these two materials. Furthermore, the types and amounts of functional groups on the surfaces of both matrices were determined using Fourier transform infrared spectroscopy (FTIR).

## Materials and methods

### Sample collection

**Fig 1:**
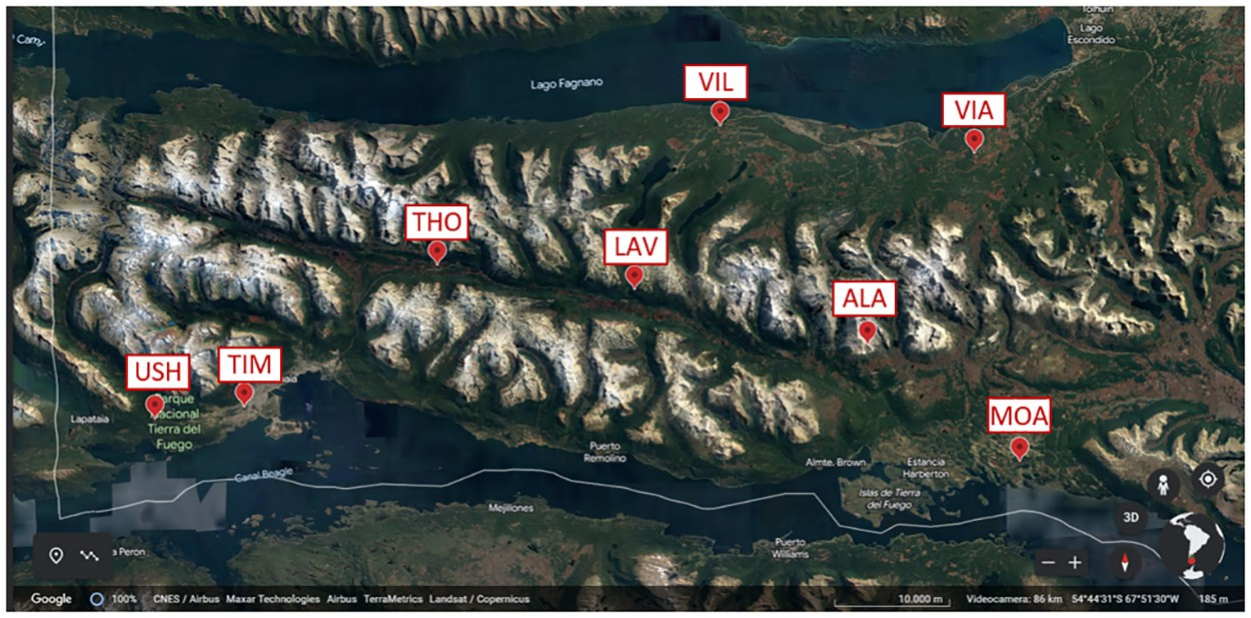
Location map of sampling sites in Tierra del Fuego (Argentina, South America). Data map: Google, CNES/Airbus, Maxar Technologies, Airbus, TerraMetrics, Landsat/Copernicus.

Eight samples (three replicates) of both ombrotrophic and mostly undisturbed peat and *Sphagnum* moss were obtained from the Tierra del Fuego (TdF), an archipelago located at the southern tip of the continent of South America. The location map of sampling sites is shown in Fig 1. As previously reported by Conti et al. [43] and Astolfi et al. [44], the sampling sites were the following: Ushuaia (USH; 54°50’2.44"S, 68°28’19.55"W), capital of Tdf and world’s southernmost city, Tierra Major (TIM; 54°49’30.44"S, 68°21’1.37"W), Tolhuin (THO; 54°42’52.82"S 68° 5’9.17"W), Laguna Victoria (LAV; 54°44’31.47"S, 67°50’9.12"W), Alambique (ALA; 54°48’33.37"S 67°31’47.63"W), Estancia Moat (MOA; 54°52’3.05"S, 67°17’32.37"W), Villa Marina (VIL; 54°36’20.77"S, 67°42’9.35"W), and Vialidad (VIA; 54°37’34.86"S, 67°21’28.27"W). Except for USH, which is the most populated in the archipelago and located near the international airport, the other sites were areas of low anthropogenic impact. All field operations were carried out in accordance with Sapienza Ethical Code–D.R. no. 1636, no. 0032773, 23/05/2012. The samples were collected in plastic bags at a distance of ~20 km from each other at a depth of 0 and 20 cm depth for *Sphagnum* moss and peat, respectively. All samples were dried in an electric stove for two days at 40 °C [24], crushed, and passed through a 2 mm sieve. All samples were stored at -20 °C until analysis.

### Metal leaching and adsorption experiments

The pH level affects the availability of some plant nutrients. Generally, different plant species adapt to soils with different pH ranges. For many species, the suitable soil pH range is relatively well known. The close relationship between the plant species and the soil pH allows you to choose the type of plant best suited to the environment and make any corrections or changes to the soil. The leachable fraction of some elements was analyzed to assess the most mobile elements, which are more easily accessible to plants and generally can be more associated with anthropogenic sources. For the element leaching and binding tests, considering precipitation phenomena for different elements at pH>5 [45], two different acidic pH (2 and 5) were considered to evaluate the availability of 39 elements (Al, As, B, Ba, Be, Bi, Ca, Cd, Ce, Co, Cr, Cs, Cu, Fe, Ga, K, La, Li, Mg, Mn, Mo, Nb, Ni, P, Pb, Rb, Sb, Se, Si, Sn, Sr, Te, Ti, Tl, U, V, W, Zn, and Zr), and adsorption of essential or beneficial trace metals for plants (Co, Cu, Fe, Mn, Ni, V, and Zn) and some non essential elements for plants or toxic or potentially toxic elements for humans (Al, As, Be, Cd, Cr, Ni, Pb, Sb, and Tl). Weighed amounts (~0.05 g) of the powder samples were transferred into 10 mL graduated polypropylene tubes (Artiglass, Due Carrare, PD, Italy) and then brought to a volume of 5 mL using deionized water (resistivity of 18.2 MΩ cm^−1^) for element leaching experiments or synthetic multi-element solution (containing Al, As, Be, Cd, Co, Cr, Cu, Fe, Mn, Ni, Pb, Sb, Tl, V, and Zn at the concentration of 10 mg/L, prepared by multi-element standard solution at 1000 mg/L from Merck, Darmstadt, Germany) for element binding test under controlled pH conditions (at pH 2 and pH 5). The pH was controlled using a Crison MicropH 2002 pH meter (Crisonb Instruments, Barcelona, Spain) and adjusted using 1% HNO_3_ (67% suprapure, Carlo Erba Reagents, Milan, Italy) and 5% NaOH (Merck, Darmstadt, Germany). All the tubes were then left under mechanical stirring at 21 °C for 24 h. The obtained solutions were filtered, diluted 1:2 with deionized water and analyzed by inductively coupled plasma mass spectrometry (ICP-MS). Method blanks are solutions made up of reagents only, which are treated and analyzed like samples to track and control the contributions from each analytical procedure and the materials used. The average values obtained from the analysis of ten method blanks are subtracted from all the results of the analyzed samples. Method blanks were also analyzed to check for any cross-contamination. The limits of determination and quantification (LOD and LOQ, respectively), as the analyte concentration corresponding to three and ten times the standard deviation of the method blanks (n = 10), are shown in S1 Table in S1 File.

### Estimation of antioxidant activity

The 2,2-diphenyl-1-picrylhydrazyl (DPPH) use is a quick, easy, and affordable method for the assessment of antioxidant properties [34–37]. The free-radical DPPH interacts with an odd electron to yield a strong absorbance at 517 nm, i.e., a purple hue. Discoloration occurs as absorbed electrons increases, leading to a yellow hue. The DPPH assay was performed as described by Frezzini et al. [46] and Astolfi et al. [47], with slight modifications. Briefly, each sample (~5 mg) was mixed with 1 mL of methanol (Merck KGaA, Darmstadt, Germany), and the mixture was shaken by rotating agitation (60 rpm, Rotator, Glas-Col, USA) for 30 min. After extraction, the solutions were filtered through a polytetrafluoroethylene (PTFE) syringe filter (Fulltech Instruments, Rome, Italy). For the DPPH assay, 50 μL of the extracted sample was added to 2 mL of methanolic DPPH (0.1 mM), and the mixture was stirred for 30 min by rotating agitation at room temperature in the dark and analyzed by UV-Vis spectrophotometry (Varian Cary 50 Bio UV-Vis, Varian Inc., Palo Alto, CA, USA) set at 517 nm by measuring the decrease in absorbance of the sample against the control (blank solution). Solutions were prepared daily and used fresh, and three replicates of each peat sample were analyzed.

**Fig 2:**
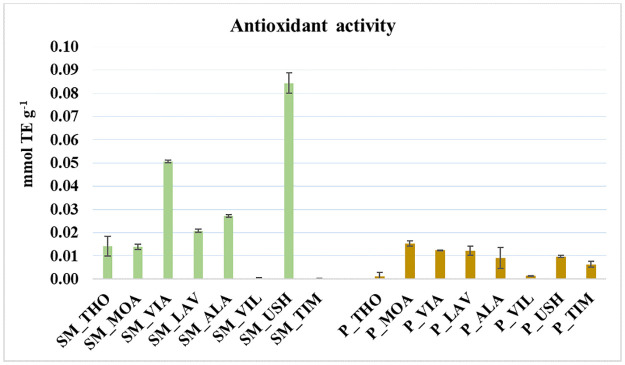
Comparison of the antioxidant capacity of *Sphagnum* moss (SM) and peat (P) using the DPPH test and Trolox standard curve. Results are presented as mean ± standard deviation (n = 16, replicates = 3).

The DPPH radical scavenging activity (RSA) results in decolorization. It was expressed as the equivalent antioxidant capacity of 6-hydroxy-2,5,7,8-tetramethylchroman-2-carboxylic acid (Trolox; Merck KGaA, Darmstadt, Germany) in mmol per gram of sample (mmol TE g^-1^), preparing a Trolox calibration curve (R^2^ = 0.99) in the range of 0.02–2.00 mM (Fig 2).

### Determination of the total phenolic level

**Fig 3:**
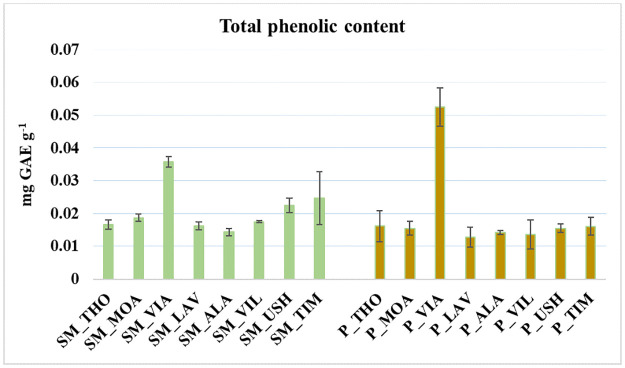
Comparison of the total phenolic content in *Sphagnum* moss (SM) and peat (P) using the gallic acid standard curve. Results are presented as mean ± standard deviation (n = 16, replicates = 3).

The total phenolic level was determined according to the Rodríguez-Flores method, with minor modifications [48]. In detail, 250 μL of the peat and *Sphagnum* moss samples extracted with 1 mL of methanol, as described in the previous section, were mixed with 2.5 mL of deionized water (produced by the Arioso UP 900 Integrate Water Purification System, USA) and 250 μL of Folin–Ciocalteu’s phenol reagent (Merck KGaA, Darmstadt, Germany). After vigorously stirring the reaction mixture for 2 min, 1 mL of 5% sodium carbonate (Merck KGaA, Darmstadt, Germany) was added. The absorbance at 765 nm was determined after 1 h by UV-Vis spectrophotometry. Gallic acid (0.1–0.5 mg mL^-1^; Sigma-Aldrich Co., St. Louis, MO, USA) was used as a standard to obtain the calibration curve (R^2^ = 0.99, Fig 3). Total phenolic concentration was expressed as mg of gallic acid equivalent per gram of dry sample (mg GAE g^-1^).

### FTIR analysis

Representative sub-samples of peat and *Sphagnum* moss were finely milled before FTIR (IR Affinity Miracle 10, Shimadzu Scientific Instruments, Columbia, MD, USA) analysis to identify the amounts and types of functional groups [44]. Spectra were acquired by averaging 200 scans at 5 cm^-1^ resolution over the 4000–600 cm^-1^.

### Statistical analysis

The data were statistically processed with IBM SPSS Statistics 27 software (IBM Corporation, Armonk, NY, USA). Spearman’s correlation analysis was carried out to examine relationships among the elemental leachable levels and correlate the peat and *Sphagnum* moss samples’ antioxidant potential and phenolic content. Statistical significance was set at p <0.05.

Multivariate statistical analysis was performed using the Chemometric Agile Tool (CAT) statistical software [49] based on the R-project for statistical computing (Ver. 3.0, 32 bits). A principal component analysis (PCA) was performed on data obtained by FTIR analysis of the 16 peat and *Sphagnum* moss samples collected at the eight different sites to group the different samples depending on the types and amounts of functional groups on their surfaces. The data matrix was transformed by centring the mean column and autoscaling the row and column before performing the PCA [50, 51].

## Results and discussion

### Leachable elements

Some authors [52, 53] used sequential digestion to discriminate between anthropogenic and natural sources of metals. This is related to the fact that weak acid leaching is assumed to release mobile elements, often regarded as anthropogenic metals. In contrast, the residual fraction is regarded as metals in the silicate fraction. Considering the results for the leachable fraction (Tables 1 and 2, and S1 Table in S1 File), many more elements differ significantly between peat and *Sphagnum* moss (Ca, Cd, Co, Ga, Rb, and Zr at both pH; Be, Bi, Ce, Cu, Fe, K, Li, Mn, P, Pb, Ti, Tl, V, and Zn at pH = 5, and Cr, Cs, Mg, Sr, and Ti at pH = 2). Peat concentrations are generally higher than in *Sphagnum* moss at pH = 2, except Cs, Rb, Ti, and Zr. In contrast, at pH = 5 all element levels in peat were lower than those in *Sphagnum* moss. Some elements (Al, As, B, Be, K, Li, Mo, Nb, P, Sb, Se, Si, Sn, Te, W, Zn, and Zr) are poorly leachable, and their concentrations are low or even undetectable. However, all concentrations of leached elements are well below the limits set by the EU Fertilizing Products Regulation (EU) 2019/1009 [54]. The release of elements in solution depends on whether they are more soluble in an acidic aqueous solution or strongly bound to the organic matrix. As highlighted by Bozau et al. [55], Pb mainly tends to form organic complexes at low pH values. Peat bogs typically have pH values below four, and the organic matter’s carboxyl groups act as a buffer system [55]. Instead, other elements (such as Si and Zr) that represent dust deposition are supposed to be immobile [55]. On the other hand, the lower leaching of elements in *Sphagnum* moss could depend on the higher presence of humic acids that are not soluble in water at low pH but become soluble under higher pH values [56, 57]. In addition, soil organic matter, unlike that in *Sphagnum* moss, is a continuum of progressively decomposing organic compounds.

**Table 1:** Results of the leachable elemental content [mean and standard deviation (SD); mg/kg d.w.; n = 8 for each material, replicates = 3] at pH = 2 in peat and *Sphagnum* moss by inductively coupled plasma mass spectrometry (ICP-MS).

Element	Peat						*Sphagnum* moss						p^c^
	n>LOD (%)	Mean	SD	Median	Min	Max	n>LOD (%)	Mean	SD	Median	Min	Max	
**Al**	0	<2	-	<2	<2	3	0	<2	-	<2	<2	<2	-
**As**	12	<0.2	-	<0.2	<0.2	0.47	28	<0.2	-	<0.2	<0.2	0.8	-
**B**	0	<1	-	<1	<1	<1	14	1	2	<1	<1	5	-
**Ba**	100	11.0	9.7	6.4	<2	27.6	100	4.6	2.1	4.2	<2	8.2	ns
**Be**	25	0.006	0.009	<0.003	<0.003	0.035	100	0.010	0.006	0.009	<0.003	0.021	-
**Bi**	25	0.0005	0.0009	<0.0004	<0.0004	0.0036	100	0.0054	0.0037	0.0044	0.0010	0.0113	ns
**Ca**	100	1336	870	**846**	494	2797	100	512	450	**304**	154	1412	**
**Cd**	100	0.16	0.28	**0.04**	<0.03	1.08	0	<0.03	-	**<0.03**	<0.03	<0.03	***
**Ce**	100	0.87	0.93	0.49	0.05	2.76	100	0.54	0.37	0.41	0.10	1.26	ns
**Co**	100	0.66	0.93	**0.31**	0.19	3.08	100	0.23	0.22	**0.18**	0.08	0.78	**
**Cr**	38	<0.02	-	**<0.02**	<0.02	0.04	100	0.23	0.21	**0.15**	0.06	0.70	***
**Cs**	88	0.0040	0.0031	**0.0035**	<0.0005	0.0097	100	0.0123	0.0091	**0.0098**	0.0034	0.0292	**
**Cu**	100	0.64	0.28	0.52	0.30	1.15	100	0.60	0.34	0.51	0.20	1.19	ns
**Fe**	100	727	740	444	249	2604	100	407	298	351	97	1069	ns
**Ga**	56	0.013	0.018	**<0.002**	<0.002	0.060	100	0.081	0.045	**0.080**	0.018	0.170	***
**K**	19	<40	-	<40	<40	70	14	<40	-	<40	<40	48	-
**La**	100	0.44	0.41	0.28	0.03	1.21	100	0.24	0.18	0.20	0.05	0.62	ns
**Li**	12	0.02	0.05	<0.01	<0.01	0.20	100	0.04	0.03	0.04	<0.01	0.10	-
**Mg**	100	809	417	**923**	153	1461	100	302	206	**259**	45	685	*
**Mn**	100	39	72	16	2	223	100	13	20	6	1	61	ns
**Mo**	0	<0.02	-	<0.02	<0.02	<0.02	100	0.08	0.11	0.04	<0.02	0.35	-
**Nb**	0	<0.002	-	<0.002	<0.002	<0.002	100	0.043	0.032	0.037	0.013	0.114	-
**Ni**	56	0.43	0.61	0.20	<0.1	2.00	100	0.26	0.14	0.22	<0.1	0.53	ns
**P**	12	<6	-	<6	<6	27	100	58	48	37	18	156	-
**Pb**	100	0.33	0.20	0.33	0.02	0.67	100	0.19	0.11	0.17	0.04	0.40	ns
**Rb**	100	0.084	0.029	**0.083**	0.045	0.134	100	0.167	0.080	**0.183**	0.067	0.315	**
**Sb**	0	<0.1	-	<0.1	<0.1	<0.1	0	<0.1	-	<0.1	<0.1	<0.1	-
**Se**	0	<0.2	-	<0.2	<0.2	<0.2	100	<0.2	-	<0.2	<0.2	0.2	-
**Si**	0	<70	-	<70	<70	<70	57	117	95	97	<70	302	-
**Sn**	0	<0.01	-	<0.01	<0.01	<0.01	57	0.015	0.008	0.016	<0.01	0.030	-
**Sr**	100	18.7	6.6	**18.5**	10.9	33.8	100	7.0	4.5	**5.7**	2.9	17.4	***
**Te**	0	<0.01	-	<0.01	<0.01	<0.01	0	<0.01	-	<0.01	<0.01	<0.01	-
**Ti**	100	4.0	1.9	**4.6**	0.4	6.6	100	20	15	**18**	3	53	***
**Tl**	0	<0.003	-	<0.003	<0.003	<0.003	57	0.0043	0.0047	<0.003	<0.003	0.0147	-
**U**	100	0.008	0.004	0.008	0.003	0.015	100	0.0158	0.0083	0.0140	0.0038	0.0281	ns
**V**	100	0.92	0.53	**0.87**	0.24	1.97	100	0.55	0.35	**0.53**	0.17	1.28	*
**W**	12	<0.02	-	<0.02	<0.02	0.20	43	<0.02	-	<0.02	<0.02	<0.02	-
**Zn**	38	<5	-	<5	<5	6.5	0	<5	-	<5	<5	<5	-
**Zr**	100	0.037	0.026	**0.026**	0.007	0.087	100	3.9	1.7	**3.9**	1.5	6.3	***

^a^LOD, limit of determination

^b^LOQ, limit of quantification

^c^Non-parametric Mann Whitney test was applied: “-”= not determined; “ns” = not significant at p >0.05;

“*” = p <0.05;

“**” = p <0.01;

“***” = p <0.001.

Numbers in bold in the same row indicate significant differences.

**Table 2:** Results of the leachable elemental content [mean and standard deviation (SD); mg/kg d.w.; (n = 8 for each material, replicates = 3] at pH = 5 in peat and *Sphagnum* moss by inductively coupled plasma mass spectrometry (ICP-MS).

Element	Peat						*Sphagnum* moss						p
	N>LOD	Mean	SD	Median	Min	Max	N > LOD	Mean	SD	Median	Min	Max	
**Al**	0	<2	-	<2	<2	28	81	9.8	7.8	8.4	<2	24.0	ns
**As**	62	0.07	-	<0.03	<0.03	0.40	68	0.19	0.25	0.09	<0.03	0.81	ns
**B**	0	<1	-	<1	<1	<1	0	<1	-	<1	<1	<1	-
**Ba**	0	<1	-	<1	<1	<1	0	<1	-	<1	<1	<1	-
**Be**	25	<0.002	-	**<0.002**	<0.002	<0.002	100	0.20	0.39	**0.06**	0.01	1.6	***
**Bi**	25	<0.0002	0.0013	<**0.0002**	<0.0002	0.0043	100	0.016	0.016	**0.011**	0.001	0.056	***
**Ca**	50	39	870	**<10**	<10	165	100	97	66	**78**	25	266	**
**Cd**	50	0.005	0.009	<**0.001**	<0.001	0.036	100	0.19	0.31	**0.09**	0.01	1.26	***
**Ce**	94	0.011	0.010	0.008	0.002	0.039	100	0.061	0.103	0.032	0.006	0.418	**
**Co**	75	0.07	0.16	**0.01**	<0.005	0.48	100	0.25	0.35	**0.16**	0.04	1.46	***
**Cr**	0	<0.1	-	<0.1	<0.1	<0.1	50	0.11	0.10	<0.1	<0.1	0.32	-
**Cs**	88	0.0015	0.0012	0.0011	<0.0001	0.0038	88	0.0025	0.0035	0.0014	<0.0001	0.0117	ns
**Cu**	94	0.094	0.088	**0.074**	<0.01	0.309	100	0.46	0.43	**0.27**	0.06	1.58	***
**Fe**	100	15	24	**4**	1	75	100	40	47	**15**	2	170	**
**Ga**	0	<0.004	-	**<0.004**	<0.004	<0.004	100	0.25	0.50	**0.06**	0.01	1.99	***
**K**	19	<40	-	**<40**	<40	178	100	279	141	**231**	89	532	***
**La**	94	0.006	0.006	0.004	<0.001	0.024	100	0.009	0.006	0.007	0.002	0.018	ns
**Li**	12	0.02	0.04	**<0.01**	<0.01	0.12	100	1.1	1.4	**0.3**	0.1	4.7	***
**Mg**	100	55	21	58	18	91	100	71	31	65	20	147	ns
**Mn**	62	8	20	**<0.1**	<0.1	60	100	10	11	**5.7**	0.3	42	***
**Mo**	0	<0.02	-	<0.02	<0.02	<0.02	44	0.23	0.56	<0.02	<0.02	1.80	ns
**Nb**	0	<0.001	-	<0.001	<0.001	<0.001	37	0.002	0.002	<0.001	<0.001	0.005	ns
**Ni**	0	<0.4	-	<0.4	<0.4	<0.4	30	<0.4	-	<0.4	<0.4	<0.4	ns
**P**	12	<6	-	**<6**	<6	45	100	72	64	**48**	17	212	***
**Pb**	0	<0.005	-	**<0.005**	<0.005	<0.005	94	0.12	0.16	**0.05**	<0.005	0.48	***
**Rb**	100	0.080	0.078	**0.045**	0.025	0.280	100	0.44	0.22	**0.36**	0.19	0.96	***
**Sb**	0	<0.2	-	<0.2	<0.2	<0.2	12	<0.2	-	<0.2	<0.2	0.3	-
**Se**	0	<0.1	-	<0.1	<0.1	<0.1	38	0.22	0.38	<0.1	<0.1	1.42	-
**Si**	0	<70	-	<70	<70	<70	0	<70	-	<70	<70	<70	-
**Sn**	0	<0.01	-	<0.01	<0.01	<0.01	30	<0.01	-	<0.01	<0.01	0.014	-
**Sr**	81	0.7	0.8	0.4	<0.3	2.6	88	0.8	0.6	0.7	<0.3	2.2	ns
**Te**	0	<0.002	-	<0.002	<0.002	<0.002	0	<0.002	-	<0.002	<0.002	<0.002	-
**Ti**	75	0.090	0.053	**0.115**	<0.04	0.164	87	0.347	0.322	**0.268**	<0.04	1.24	**
**Tl**	0	<0.001	-	**<0.001**	<0.001	<0.001	100	0.26	0.42	**0.15**	0.01	1.71	***
**U**	81	0.0006	0.0004	0.0005	<0.0003	0.0015	87	0.0016	0.0017	0.0010	<0.0003	0.0052	ns
**V**	12	<0.1	-	**<0.1**	<0.1	0.17	62	0.19	0.17	**0.20**	<0.1	0.66	***
**W**	12	<0.01	-	<0.01	<0.01	0.14	43	1.1	2.8	0.02	<0.01	9.7	ns
**Zn**	38	0.26	0.52	**<0.05**	<0.05	1.7	100	2.7	2.9	**2.1**	0.8	12.7	***
**Zr**	100	0.079	0.051	**0.056**	0.036	0.210	100	1.5	1.6	**1.1**	0.1	5.7	***

^a^LOD, limit of determination

^b^LOQ, limit of quantification

^c^Non-parametric Mann Whitney test was applied: “-”= not determined; “ns” = not significant at p >0.05;

“*” = p <0.05;

“**” = p <0.01;

“***” = p <0.001.

Numbers in bold in the same row indicate significant differences.

Considering the Spearman’s rank correlation for leachable elements at pH = 2 (S2 and S3 Tables in S1 File), there are elements whose concentrations are strongly correlated to a large number of other elements and elements that are not correlated with any (e.g. Ca, Cd, and Rb in peat and Mn in *Sphagnum* moss). Both in peat and in *Sphagnum* moss there are high correlations (from 0.7 to 1) between the following pairs of elements Ba-Ni, Ce-Fe, Ce-La, Co-Fe, Fe-La, Mg-Na and Sr-Zr, indicating a possible common origin probably of a natural type. It has been well documented that Ba, Ca, Co, Cr, Fe, Ga, Mn, Ni, Sr, Ti, V, and Zr are elements mainly lithogenic in nature, and in ombrotrophic peats, they come mainly from the deposition of dust particles released into the atmosphere by soil erosion [58]. However, anthropogenic contributions to these elements cannot be excluded. Chromium, Co, Cu, Ni, and Cd can have anthropogenic sources such as emissions from industrial production and the burning of fossil fuels [59, 60]. Copper in the surface peat layer can be associated with forming strong complexes of Cu oxides with humic acids [61]. The formation of metal-organic complexes is an important bonding process of the metal on the surface of the swamp, which could lead to the formation of soluble complexes of Cr, Fe, and Mn (e.g. short-chain organic acids or fulvic acids) [58]. Furthermore, some elements such as Cu, Cd, Fe, Mn and Ni can be influenced by changes in the redox and pH conditions with possible consequent leaching [60, 62]. Lead has several anthropogenic sources and becomes part of the composition of peat and moss due to aerosols released following various industrial productions and fuel combustion [60]. Lead has a high affinity for organic matter; for this reason, it is the most reliable indicator in retrospective studies on pollution [63–66]. Our study showed a high correlation between Pb-Cs and Pb-Mn in peat and between Pb-Ti and Pb-Cr in *Sphagnum* moss in the leached fraction, suggesting a dual origin of both natural and anthropogenic Pb. In fact, as previously reported, Cr, Mn and Ti are elements present in ombrotrophic peat mainly due to the deposition of dust particles released by soil erosion [58]. Instead, water-soluble Cs can be considered a tracer of combustion processes (mainly related to biomass combustion) [67]. However, it is important to underline that some elements typically emitted from combustion sources (As, Cd, Pb, Sb, Sn, and V) are present in the fine fraction of the atmospheric particulate as insoluble nanoparticles [68, 69].

### Element adsorption

The metal removal efficiency of peat and *Sphagnum* moss is strongly dependent on pH and metal characteristics [18, 19]. González et al. [19] showed that metal adsorption on *Sphagnum* sp. typically starts at pH around 2, and the maximum adsorption percentage is achieved at pH = 5.5, 6.1, 7.2, 7.8, and 8.7 for Pb (97%), Cu (86%), Ni (70%), Zn (73%), and Cd (91%), respectively. Also, Liu et al. [70] showed that the Cd, Cu, and Ni adsorption percentage on peat increases with the pH increase from 2 to 6. At pH <3, hydrogen ions can compete with metal ions. However, it was demonstrated by Gosset et al. [71] that Ni can bind strongly to peat even at acidic pH. At pH ≥5.5, precipitation can occur as hydroxides of various elements (such as As, Bi, Fe, Pb, and Sb), thus it was not possible to quantify their removal [45]. Our goal was to do the adsorption tests at pH = 2 and 5, which are also suitable pH values for various flowers, fruits, and vegetables. The data reported in Table 3 highlight *Sphagnum* moss’s greater adsorption capacity than peat for all elements, especially at pH = 5, except Be, Mn, Tl, and Zn. In particular, Bi and Sb are retained by *Sphagnum* moss and peat at pH = 2.

**Table 3:** Percentage of element adsorbed onto *Sphagnum* moss and peat surface as a function of pH (concentration of 10 mg/L, 24 h; n = 8 for each material and pH value, replicates = 3).

Element	*Sphagnum* moss				Peat			
	pH = 5		pH = 2		pH = 5		pH = 2	
	mean	SD	mean	SD	mean	SD	mean	SD
**Al**	43	17	<10	-	<10	-	<10	-
**As**	<10	-	<10	-	<10	-	<10	-
**Be**	53	11	10	7	59	23	<10	-
**Bi**	-	-	91	5	-	-	94	4
**Cd**	53	20	<10	-	<10	-	<10	-
**Co**	38	20	<10	-	<10	-	<10	-
**Cr**	86	5	10	6	<10	-	<10	-
**Cu**	88	5	12	7	<10	-	<10	-
**Fe**	-	-	24	17	<10	-	<10	-
**Mn**	34	20	12	8	98	2	<10	-
**Ni**	44	18	<10	-	<10	-	<10	-
**Pb**	-	-	<10	-	-	-	<10	-
**Sb**	-	-	72	7	-	-	72	7
**Tl**	31	11	21	8	88	6	12	7
**V**	57	10	<10	-	<10	-	18	25
**Zn**	44	21	<10	-	61	23	<10	-

Antimony and Tl are considered emerging contaminants and toxic for humans, animals, microorganisms, and plants [72]. Antimony mainly exists in two oxidation states, such as Sb(III) (the most toxic form) and Sb(V) [73]. In soils and groundwater, Sb(V) appears to be the predominant form [73]. The best pH range for Sb(V) removal is pH 2–5.5 when Sb(OH)6− is the predominant form. The adsorption capacity decreased at pH >5.5 due to possible charge repulsion between the negatively charged surface groups of the adsorbent and the negatively charged Sb(V) ions and competition for adsorption sites between the hydroxide and antimonate ions [74].

The maximum adsorbent capacity of Tl occurs with peat at pH 5 (Table 3). This is probably because as the pH increases from 2 to 10, the surface functional groups are deprotonated more, giving them a greater chance of binding to Tl [75]. In fact, an excess of H+ could form competitive adsorption sites with Tl [76]. In addition, at lower pH, the carbon surface transmits positive charges, which results in electrostatic repulsion force with Tl particles [75].

### Antioxidant activity and total phenolic content

Among the approaches used to study antioxidant activities is the DPPH assay, which is based on observing DPPH color changes when it reacts with the scavenger radical [25, 77]. The level of transparency of the color indicates the intensity of the antioxidant activity. In the DPPH assay, the moss samples showed a higher overall antioxidant capacity (from 0.3 ± 0.1 μmol TE g^-1^ to 84.4 ± 4.4 μmol TE g^-1^) than peat (from 1.3 ± 1.7 μmol TE g^-1^ to 15.3 ± 1.1 μmol TE g^-1^) (Fig 2). Additionally, the *Sphagnum* moss samples showed significantly higher mean antioxidant activity (26.2 ± 28.4 μmol TE g^-1^) than that estimated in peat (9.4 ± 5.1 μmol TE g^-1^). Some studies have described the possibility of using *Sphagnum* moss as a source of dietary fiber and functional food [77, 78]. This study agreed with other studies [77] that Patagonian *Sphagnum* moss has antioxidant properties. However, the antioxidant activity value of *Sphagnum* moss is lower than that found in plant species intended for human consumption, such as broccoli with 10.2–23.5 μmol TE g^-1^ [79, 80] and garlic with 7.4–11.9 μmol TE g^-1^ [81], and fruit, peel, and pulp of citrus fruits ranging from 12.2 (lemon pulp) to 444 μmol TE g^-1^ (mandarin peel) [82].

In the present study, peat and *Sphagnum* moss samples have a similar amount of total phenolic content [0.018 ± 0.011 mg GAE g^-1^ for peat and 0.020 ± 0.007 mg GAE g^-1^ for *Sphagnum* moss] (Fig 3). Some studies [83–85] showed that phenolic compounds can contribute to antioxidant activity thanks to hydroxyl groups, which can eliminate free radicals and reactive oxygen species [86]. In contrast, our results do not show a significant correlation between total phenol concentration and antioxidant activity in both matrices (p >0.05). Therefore, it can be assumed that inhibition of DPPH radical scavenging by peat and *Sphagnum* moss was not strictly proportional to total phenol concentration but was related to the different amounts and types of surface functional groups that interact more with free radicals, among which phenolic groups.

The FTIR spectra of peat and *Sphagnum* moss samples in the range of 4000–600 cm^−1^ are shown in S1 Fig. As described in a previous work [44], the spectra are characterized by broad bands, which are typical for natural organic matter due to the overlap of individual absorption bands [87]. Generally, characteristic bands are recognized in a specific region of the spectrum [88, 89]. For example, a broad band between 3000 and 3700 cm^-1^ can be observed due to the OH stretching of various groups such as alcohols and phenols in cellulose [89, 90]. This region is greater in *Sphagnum* moss, whereas it appears to have been reduced in peat. The absorption bands at approximately 2850 and 2920 cm^−1^ are characteristic of CH groups in aliphatic compounds, which are ascribed to lipids of plant, bacterial, or fungal origin [88, 89]. Spectral bands indicative of lignin (such as 1513, 1450, 1371, 1265, and 835 cm^−1^) include contributions from the vibrations of aromatic C = C stretching, C–H deformation, and C–O stretching of phenolic OH and/or arylmethylethers [88, 89]. Aromatic and/or aliphatic carboxylates (humic acids) show bands in the 1650–1600 and 1426 cm^−1^ regions due to aromatic C = C stretching and/or asymmetric C–O stretching in COO–(R-COO–) or stretching and OH deformation (COOH) [88]. The FTIR spectra of the fulvic acids showed a more pronounced adsorption band in the 1720 cm^−1^ region, indicating a larger content of carboxyl groups [91]. The bands of inorganic matter mainly correspond to those of silicon and clay minerals (~780 cm^−1^) and silicon and polysaccharides (910–1080 cm^−1^) [91].

**Fig 4:**
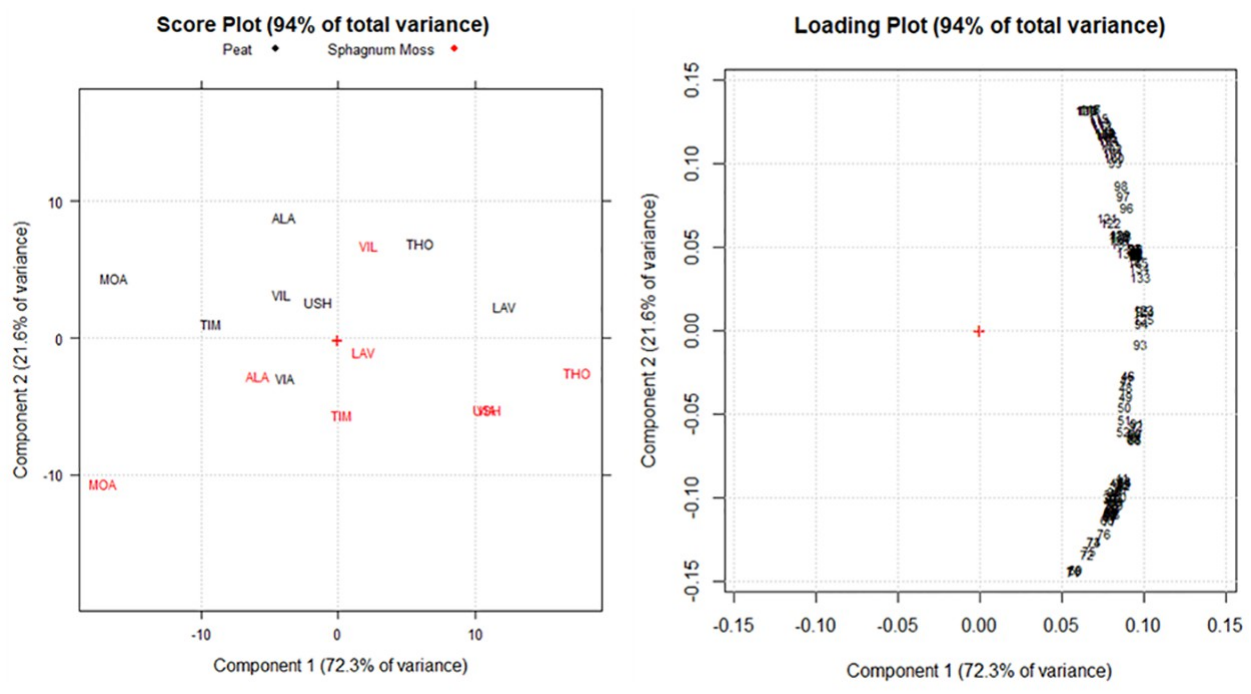
PCA score plot and loading plot performed on the obtained FTIR spectral data of the peat and *Sphagnum* moss samples (n = 8 for each material).

**Fig 5:**
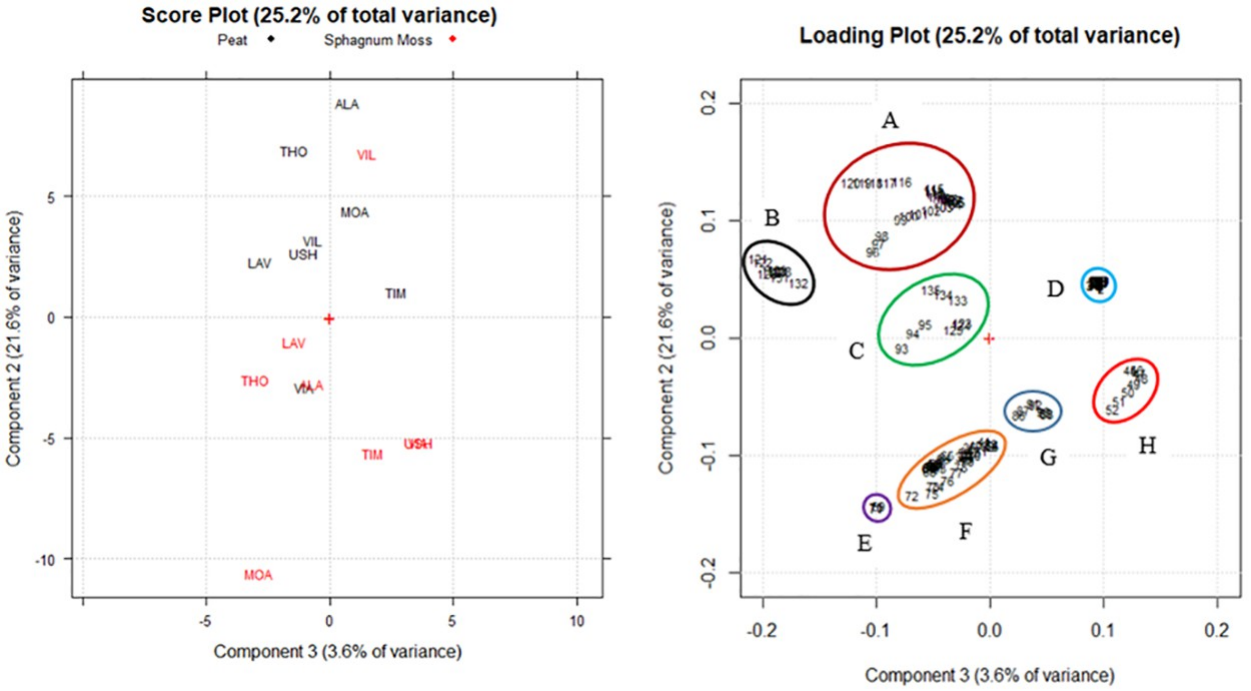
PCA score plot and loading plot performed on the obtained FTIR spectral data of the peat and *Sphagnum* moss samples (n = 8 for each material). A. Amide III, carbohydrates, aromatic ethers, Si-O-C groups, Si-O stretching, B. Clay minerals, kaolinite doublet, smectite, clays, quartz, C. Polysaccharides, alcoholic groups, clays, quartz, D. Cellulose, E. Proteinaceous origin, F. Lignin/phenolic backbone, phenolic (lignin) and aliphatic structures, carboxylate, carboxylic structures (humic acids), G. Lignin, secondary amides, H. Carboxylic acids, aromatic esters.

By PCA of the spectral data by FTIR analysis of the 16 peat and *Sphagnum* moss samples, five significant components accounting for 99.5% were obtained. The variance explained by each component was 72.3, 21.6, 3.6, 1.6, and 0.3%, respectively. The first component (PC1), which explained 72.3% of the total variance (Fig 4), separated the samples (scores) mainly depending on the multiplicative variations between the spectra caused by variations in the sample’s physical properties or sample preparation and presentation. Therefore, the score and loading plots of PC2/PC3 (25.2% of the total variance, Fig 5), which differentiate the samples exclusively depending on the different amounts and types of functional groups on their surfaces, are presented here.

Fig 5 shows that the peat and *Sphagnum* moss samples were grouped into eight main clusters (marked in different colors) on PC2 and PC3 of the score plot. In general, PC2 separates *Sphagnum* moss samples (except the VIL site sample) from peat samples (except the VIA site sample) depending on the different functional group contents of clays, silicates, and quartz, which are more commonly present in peat than in *Sphagnum* moss. On the contrary, the functional groups of phenolics, humic acids, carboxylic acids, proteins, and lignin were considerably more abundant in the samples of *Sphagnum* moss. The alcohol and polysaccharide groups did not seem to vary within the entire dataset, with no variation between peat and *Sphagnum* moss. On the other hand, PC3 differentiates the peat and *Sphagnum* moss samples based on the functional groups most present at the different sites where the sample was collected.

Concerning the peat samples, at the LAV site, there is a higher content of clay and quartz groups and at the ALA site, there is a higher content of SiO and SiOC. In contrast, for the *Sphagnum* moss, there are greater groups of lignin and proteins in the samples taken at the MOA site. In samples from the USH and VIA sites, the highest content of phenolic groups, humic acids, and carboxylic acids (i.e., of the groups in the clusters marked in red and blue) were identified as the functional groups that determine the antioxidant activity of *Sphagnum* moss (Fig 2).

## Conclusions

Many more elements differ significantly between peat and *Sphagnum* moss. At pH 2, peat generally has higher concentrations of leachable elements compared to *Sphagnum* moss, except for Cs, Rb, Ti, and Zr. On the other hand, at pH 5, the concentration of leachable elements in peat is lower than in *Sphagnum* moss. Generally, several elements (Al, As, B, Be, K, Li, Mo, Nb, P, Sb, Se, Si, Sn, Te, W, Zn, and Zr) are poorly leachable, with low or undetectable concentrations. However, all concentrations of leached elements are well below the limits set for fertilizers by the EU Fertilizing Products Regulation (EU) 2019/1009. For the element adsorption it is noteworthy that the adsorption capacity of both materials is pH-dependent, with higher adsorption at pH 5 compared to pH 2. *Sphagnum* moss generally exhibits greater adsorption capacity than peat for most elements at both pH levels. Given *Sphagnum* moss’s superior adsorption capacity, it can be more effectively utilized for environmental cleanup and remediation, particularly in areas with varying pH levels. Further research should explore optimizing conditions for maximum adsorption efficiency, considering the specific pH requirements of different pollutant. The antioxidant properties of peat and *Sphagnum* moss are important parameters that can influence the oxidative degradation of organic matter. The total phenolic content determined in our study was similar in peat and *Sphagnum* moss, even though a higher DPPH assay was observed in *Sphagnum* moss. The results of this study suggest that the phenolic, humic acid, and carboxylic acid groups are the components that mostly determine the antioxidant activity of *Sphagnum* moss.

This study unequivocally supports the hypothesis that our data serve as a baseline for informing management decisions regarding future environmental protection/prevention programs.

## Supporting information

S1 Fig FTIR spectra of peat (A) and *Sphagnum* moss (B) samples (n = 8 for each material). (TIF)

S1 File (DOCX)
