# Supplementary figures and images for: Sphagnum moss and peat comparative study: Metal release, binding properties and antioxidant activity
Source: PLoS One. 2024 Aug 19;19(8):e0307210. doi: 10.1371/journal.pone.0307210 (PMC11332952; doi:10.1371/journal.pone.0307210)

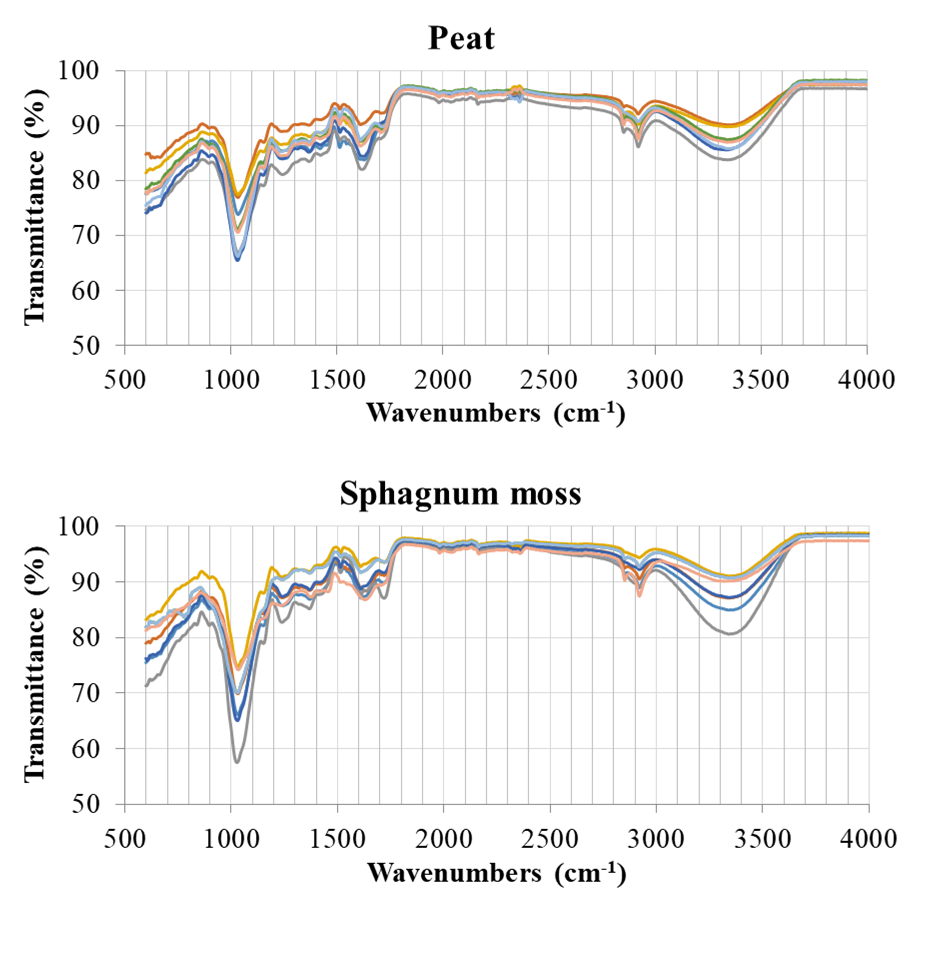

Supplement: S1 Fig — (TIF) [file pone.0307210.s001.tif]
