# Supplementary material for: Sphagnum moss and peat comparative study: Metal release, binding properties and antioxidant activity
Source: PLoS One. 2024 Aug 19;19(8):e0307210. doi: 10.1371/journal.pone.0307210 (PMC11332952; doi:10.1371/journal.pone.0307210)
Supplement: S1 File — (DOCX) [file pone.0307210.s002.docx]

**Supporting information**

*Sphagnum* moss and peat comparative study: metal release, binding properties and antioxidant activity

**Maria Luisa Astolfi** **^1,2,*^**, **Maria Agostina Frezzini** **^3^**, **Lorenzo Massimi** **^4,5^**, **Mattia Rapa** **^6^**, **Silvia Canepari** **^4,5^**, **Marcelo Enrique Conti ^6^**

^1^ Department of Chemistry, Sapienza University of Rome, Rome, Italy

^2^ Research Center for Applied Sciences to the Safeguard of Environment and Cultural Heritage (CIABC), Sapienza University of Rome, Rome, Italy

^3^ ARPA Lazio, Regional Environmental Protection Agency, Rome, Italy

^4^ Department of Environmental Biology, Sapienza University of Rome, Rome, 00185, Italy

^5^ C.N.R. Institute of Atmospheric Pollution Research, Monterotondo St., Rome, Italy

^6^ Department of Management, Sapienza University of Rome, Rome, Italy

^*^Corresponding author

Email: marialuisa.astolfi@uniroma1.it

| 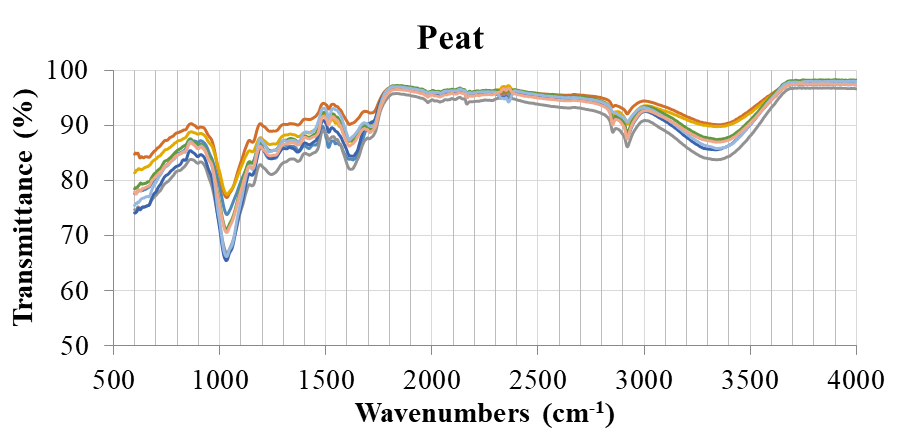  **A** |
| --- |
| **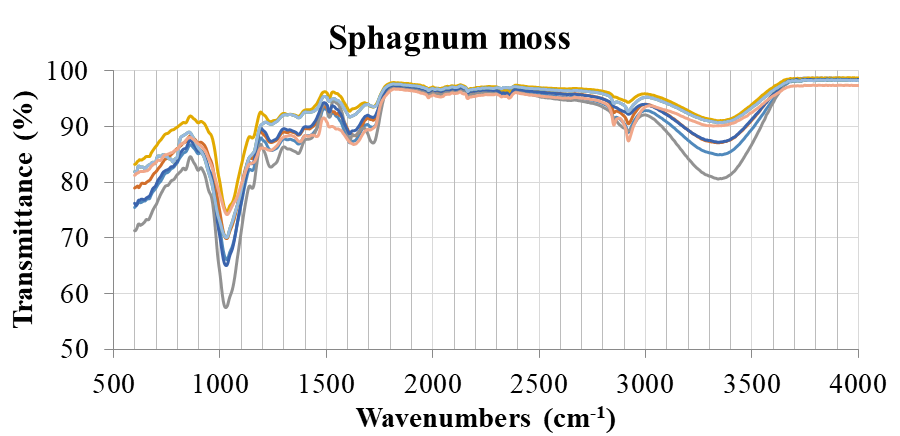**  **B** |

**Figure S1. FTIR spectra of peat (A) and *Sphagnum* moss (B) samples (n = 8 for each material).**

**Table S1. Limit of determination and quantification (mg/kg) for each element at pH 5 and pH 2 (n = 10 for each pH value).**

|  | **pH = 5** | | **pH = 2** | |
| --- | --- | --- | --- | --- |
| **Element** | **LOD** | **LOQ** | **LOD** | **LOQ** |
| **Al27** | 0.4 | 1 | 2 | 8 |
| **As75** | 0.03 | 0.09 | 0.2 | 0.7 |
| **B11** | 1 | 5 | 1 | 4 |
| **Ba137** | 1 | 2 | 2 | 5 |
| **Be9** | 0.002 | 0.006 | 0.003 | 0.01 |
| **Bi209** | 0.0002 | 0.0007 | 0.0004 | 0.001 |
| **Ca44** | 10 | 40 | 100 | 400 |
| **Cd112** | 0.001 | 0.005 | 0.03 | 0.09 |
| **Ce140** | 0.0003 | 0.001 | 0.01 | 0.05 |
| **Co59** | 0.005 | 0.02 | 0.02 | 0.07 |
| **Cr52** | 0.1 | 0.2 | 0.02 | 0.06 |
| **Cs133** | 0.0001 | 0.0004 | 0.0005 | 0.002 |
| **Cu65** | 0.01 | 0.04 | 0.06 | 0.2 |
| **Fe56** | 0.3 | 0.9 | 1 | 3 |
| **Ga71** | 0.004 | 0.01 | 0.002 | 0.01 |
| **K39** | 60 | 200 | 40 | 150 |
| **La139** | 0.001 | 0.004 | 0.001 | 0.003 |
| **Li7** | 0.01 | 0.03 | 0.01 | 0.04 |
| **Mg24** | 7 | 20 | 3 | 8 |
| **Mn55** | 0.1 | 0.2 | 0.01 | 0.03 |
| **Mo98** | 0.02 | 0.07 | 0.02 | 0.08 |
| **Nb93** | 0.001 | 0.003 | 0.002 | 0.008 |
| **Ni60** | 0.4 | 1 | 0.1 | 0.4 |
| **P31** | 8 | 30 | 6 | 20 |
| **Pb208** | 0.005 | 0.02 | 0.005 | 0.02 |
| **Rb85** | 0.01 | 0.02 | 0.01 | 0.02 |
| **Sb121** | 0.2 | 0.7 | 0.1 | 0.2 |
| **Se76** | 0.1 | 0.2 | 0.2 | 0.8 |
| **Si28** | 60 | 200 | 70 | 200 |
| **Sn118** | 0.008 | 0.03 | 0.01 | 0.04 |
| **Sr88** | 0.3 | 1 | 0.1 | 0.4 |
| **Te125** | 0.002 | 0.007 | 0.01 | 0.04 |
| **Ti49** | 0.04 | 0.1 | 0.05 | 0.2 |
| **Tl205** | 0.001 | 0.003 | 0.003 | 0.01 |
| **U238** | 0.0003 | 0.001 | 0.0003 | 0.001 |
| **V51** | 0.1 | 0.2 | 0.02 | 0.05 |
| **W182** | 0.01 | 0.04 | 0.02 | 0.07 |
| **Zn66** | 0.05 | 0.2 | 5 | 20 |
| **Zr90** | 0.002 | 0.008 | 0.003 | 0.009 |

**Table S2. Spearman correlation coefficients (r) between all leachable elements in peat (n = 8, replicates = 3).**

|  | **Na** | **Mg** | **Ti** | **Co** | **Ni** | **Cu** | **Ga** | **Rb** | **Sr** | **Zr** | **Cd** | **Cs** | **Ba** | **La** | **Ce** | **Pb** | **U** | **Ca** | **V** | **Cr** | **Mn** |
| --- | --- | --- | --- | --- | --- | --- | --- | --- | --- | --- | --- | --- | --- | --- | --- | --- | --- | --- | --- | --- | --- |
| **Mg** | **,940^**^** | -- |  |  |  |  |  |  |  |  |  |  |  |  |  |  |  |  |  |  |  |
| **Ti** | -0.128 | -0.085 | -- |  |  |  |  |  |  |  |  |  |  |  |  |  |  |  |  |  |  |
| **Co** | -,625^**^ | **-,726^**^** | 0.262 | -- |  |  |  |  |  |  |  |  |  |  |  |  |  |  |  |  |  |
| **Ni** | -,539^*^ | -,691^**^ | 0.212 | **,771^**^** | -- |  |  |  |  |  |  |  |  |  |  |  |  |  |  |  |  |
| **Cu** | -0.427 | -,538^*^ | -0.191 | ,606^*^ | ,550^*^ | -- |  |  |  |  |  |  |  |  |  |  |  |  |  |  |  |
| **Ga** | -0.316 | -0.215 | ,553^*^ | 0.166 | 0.215 | 0.025 | -- |  |  |  |  |  |  |  |  |  |  |  |  |  |  |
| **Rb** | -0.068 | -0.165 | 0.179 | 0.312 | 0.353 | 0.429 | -0.166 | -- |  |  |  |  |  |  |  |  |  |  |  |  |  |
| **Sr** | **,720^**^** | ,679^**^ | -0.144 | -0.297 | -0.479 | -0.026 | 0.009 | 0.003 | -- |  |  |  |  |  |  |  |  |  |  |  |  |
| **Zr** | -,677^**^ | **-,724^**^** | 0.326 | ,526^*^ | ,608^*^ | 0.444 | 0.154 | 0.203 | **-,712^**^** | -- |  |  |  |  |  |  |  |  |  |  |  |
| **Cd** | -0.138 | -0.162 | -0.135 | -0.112 | -0.129 | 0.200 | 0.083 | -0.121 | 0.159 | 0.085 | -- |  |  |  |  |  |  |  |  |  |  |
| **Cs** | 0.296 | 0.443 | ,690^**^ | -0.334 | -0.462 | -,583^*^ | 0.249 | -0.130 | 0.119 | -0.109 | -0.182 | -- |  |  |  |  |  |  |  |  |  |
| **Ba** | -0.375 | -,535^*^ | 0.071 | **,750^**^** | **,811^**^** | ,632^**^ | 0.080 | 0.403 | -0.171 | 0.344 | 0.082 | -,605^*^ | -- |  |  |  |  |  |  |  |  |
| **La** | **-,764^**^** | **-,774^**^** | 0.412 | **,832^**^** | **,737^**^** | ,615^*^ | ,540^*^ | 0.132 | -0.376 | ,656^**^ | 0.076 | -0.175 | ,632^**^ | -- |  |  |  |  |  |  |  |
| **Ce** | **-,781^**^** | **-,735^**^** | 0.382 | **,829^**^** | **,715^**^** | ,503^*^ | 0.479 | 0.150 | -0.432 | ,541^*^ | -0.088 | -0.166 | ,615^*^ | **,950^**^** | -- |  |  |  |  |  |  |
| **Pb** | 0.093 | 0.118 | ,674^**^ | -0.141 | -0.279 | -0.338 | 0.144 | -0.076 | -0.044 | 0.194 | 0.088 | **,834^**^** | -0.456 | -0.024 | -0.135 | -- |  |  |  |  |  |
| **U** | -,640^**^ | **-,774^**^** | 0.329 | **,941^**^** | **,838^**^** | ,647^**^ | 0.107 | 0.379 | -0.429 | ,691^**^ | -0.044 | -0.311 | **,788^**^** | **,841^**^** | **,797^**^** | -0.032 | -- |  |  |  |  |
| **Ca** | 0.240 | 0.194 | 0.068 | -0.188 | 0.104 | 0.338 | 0.335 | 0.441 | 0.338 | 0.044 | 0.253 | -0.102 | 0.185 | 0.006 | -0.135 | -0.150 | -0.115 | -- |  |  |  |
| **V** | -0.347 | -0.382 | **,762^**^** | 0.491 | ,500^*^ | 0.194 | **,777^**^** | 0.035 | -0.159 | ,518^*^ | 0.076 | 0.241 | 0.365 | ,694^**^ | ,550^*^ | 0.368 | ,526^*^ | 0.285 | -- |  |  |
| **Cr** | 0.115 | 0.179 | -0.076 | -0.279 | -0.206 | -,551^*^ | -0.039 | -0.429 | -0.191 | 0.025 | -0.201 | 0.178 | -,553^*^ | -0.343 | -0.309 | 0.105 | -0.382 | -0.262 | -0.101 | -- |  |
| **Mn** | -0.010 | -0.112 | -0.400 | 0.279 | ,510^*^ | ,521^*^ | -0.163 | 0.424 | 0.021 | 0.094 | -0.106 | **-,746^**^** | ,641^**^ | 0.135 | 0.171 | **-,829^**^** | 0.262 | 0.462 | -0.115 | -0.167 | -- |
| **Fe** | -,558^*^ | -0.494 | 0.224 | **,750^**^** | ,602^*^ | ,541^*^ | 0.430 | 0.147 | -0.191 | 0.329 | -0.247 | -0.222 | ,606^*^ | **,832^**^** | **,912^**^** | -0.341 | ,659^**^ | -0.029 | 0.409 | -0.360 | 0.353 |

^a^ Values in bold indicate strong Spearman correlation (0.7 to 1.0). The results were considered to be statistically significant with p-values of <0.05 (“*” = p <0.05; and “**” = p <0.01). When the percentage of values <limit of detection exceeded 30%, the element was excluded from the statistical elaboration (Al, As, B, Be, Bi, K, Li, Mo, Nb, P, Sb, Se, Si, Sn, Te, Tl, W, Zn).

**Table S3. Spearman correlation coefficients (r) between all leachable elements in *Sphagnum* moss (n = 8, replicates = 3).**

| **Elements^a^** | **Na** | **Mg** | **Ti** | **Co** | **Ni** | **Cu** | **Ga** | **Rb** | **Sr** | **Zr** | **Cs** | **Ba** | **La** | **Ce** | **Pb** | **U** | **Ca** | **V** | **Cr** | **Mn** |
| --- | --- | --- | --- | --- | --- | --- | --- | --- | --- | --- | --- | --- | --- | --- | --- | --- | --- | --- | --- | --- |
| **Mg** | **,808^*^** | -- |  |  |  |  |  |  |  |  |  |  |  |  |  |  |  |  |  |  |
| **Ti** | 0.394 | 0.464 | -- |  |  |  |  |  |  |  |  |  |  |  |  |  |  |  |  |  |
| **Co** | -0.335 | -0.286 | -0.321 | -- |  |  |  |  |  |  |  |  |  |  |  |  |  |  |  |  |
| **Ni** | -0.079 | -0.393 | 0.071 | 0.536 | -- |  |  |  |  |  |  |  |  |  |  |  |  |  |  |  |
| **Cu** | 0.670 | 0.536 | 0.357 | 0.071 | 0.071 | -- |  |  |  |  |  |  |  |  |  |  |  |  |  |  |
| **Ga** | 0.335 | 0.571 | **,893^**^** | -0.143 | 0.107 | 0.357 | -- |  |  |  |  |  |  |  |  |  |  |  |  |  |
| **Rb** | **,808^*^** | **,893^**^** | 0.321 | 0.000 | -0.107 | 0.429 | 0.429 | -- |  |  |  |  |  |  |  |  |  |  |  |  |
| **Sr** | 0.571 | **,857^*^** | 0.393 | 0.071 | -0.321 | 0.714 | 0.571 | 0.714 | -- |  |  |  |  |  |  |  |  |  |  |  |
| **Zr** | -0.512 | -0.679 | 0.000 | -0.321 | 0.000 | -0.643 | -0.321 | -0.679 | **-,821^*^** | -- |  |  |  |  |  |  |  |  |  |  |
| **Cs** | 0.453 | **,821^*^** | 0.500 | -0.071 | -0.250 | 0.107 | 0.679 | **,821^*^** | 0.679 | -0.500 | -- |  |  |  |  |  |  |  |  |  |
| **Ba** | -0.039 | -0.429 | 0.036 | 0.464 | **,857^*^** | 0.393 | 0.000 | -0.286 | -0.214 | -0.036 | -0.536 | -- |  |  |  |  |  |  |  |  |
| **La** | -0.493 | -0.571 | 0.036 | 0.750 | 0.679 | 0.107 | 0.036 | -0.429 | -0.179 | 0.107 | -0.393 | 0.750 | -- |  |  |  |  |  |  |  |
| **Ce** | -0.493 | -0.571 | 0.036 | 0.750 | 0.679 | 0.107 | 0.036 | -0.429 | -0.179 | 0.107 | -0.393 | 0.750 | **1,00^**^** | -- |  |  |  |  |  |  |
| **Pb** | 0.433 | 0.643 | **,857^*^** | -0.393 | -0.286 | 0.143 | 0.750 | 0.536 | 0.464 | 0.000 | 0.750 | -0.429 | -0.286 | -0.286 | -- |  |  |  |  |  |
| **U** | 0.335 | 0.143 | 0.679 | -0.036 | 0.321 | 0.750 | 0.536 | 0.000 | 0.321 | -0.107 | -0.107 | 0.607 | 0.429 | 0.429 | 0.286 | -- |  |  |  |  |
| **Ca** | 0.670 | 0.536 | 0.357 | 0.071 | 0.071 | **1,00^**^** | 0.357 | 0.429 | 0.714 | -0.643 | 0.107 | 0.393 | 0.107 | 0.107 | 0.143 | 0.750 | -- |  |  |  |
| **V** | 0.197 | 0.143 | 0.714 | 0.071 | 0.429 | 0.643 | 0.714 | 0.000 | 0.357 | -0.214 | 0.036 | 0.607 | 0.500 | 0.500 | 0.286 | **,929^**^** | 0.643 | -- |  |  |
| **Cr** | 0.512 | **,786^*^** | **,786^*^** | -0.321 | -0.143 | 0.214 | **,893^**^** | 0.679 | 0.607 | -0.357 | **,893^**^** | -0.357 | -0.357 | -0.357 | **,857^*^** | 0.214 | 0.214 | 0.357 | -- |  |
| **Mn** | 0.493 | 0.071 | -0.179 | 0.429 | 0.607 | 0.357 | -0.214 | 0.429 | 0.000 | -0.321 | -0.071 | 0.536 | 0.179 | 0.179 | -0.250 | 0.107 | 0.357 | 0.000 | -0.179 | -- |
| **Fe** | -0.335 | -0.393 | -0.357 | **,964^**^** | 0.607 | 0.179 | -0.214 | -0.143 | 0.000 | -0.286 | -0.286 | 0.643 | **,821^*^** | **,821^*^** | -0.536 | 0.107 | 0.179 | 0.179 | -0.464 | 0.464 |

^a^ Values in bold indicate strong Spearman correlation (0.7 to 1.0). The results were considered to be statistically significant with p-values of <0.05 (“*” = p <0.05; and “**” = p <0.01). When the percentage of values <limit of detection exceeded 30%, the element was excluded from the statistical elaboration (Al, As, B, Be, Bi, Cd, K, Li, Mo, Nb, P, Sb, Se, Si, Sn, Te, Tl, W, Zn).
